# Encoding Time Series as Multi-Scale Signed Recurrence Plots for Classification Using Fully Convolutional Networks

**DOI:** 10.3390/s20143818

**Published:** 2020-07-08

**Authors:** Ye Zhang, Yi Hou, Shilin Zhou, Kewei Ouyang

**Affiliations:** College of Electronic Science and Technology, National University of Defense Technology, Changsha 410073, China; zhangye18@nudt.edu.cn (Y.Z.); slzhou@nudt.edu.cn (S.Z.); ouyangkewei14@nudt.edu.cn (K.O.)

**Keywords:** time series classification, multi-scale signed recurrence plots, fully convolutional networks

## Abstract

Recent advances in time series classification (TSC) have exploited deep neural networks (DNN) to improve the performance. One promising approach encodes time series as recurrence plot (RP) images for the sake of leveraging the state-of-the-art DNN to achieve accuracy. Such an approach has been shown to achieve impressive results, raising the interest of the community in it. However, it remains unsolved how to handle not only the variability in the distinctive region scale and the length of sequences but also the tendency confusion problem. In this paper, we tackle the problem using Multi-scale Signed Recurrence Plots (MS-RP), an improvement of RP, and propose a novel method based on MS-RP images and Fully Convolutional Networks (FCN) for TSC. This method first introduces phase space dimension and time delay embedding of RP to produce multi-scale RP images; then, with the use of asymmetrical structure, constructed RP images can represent very long sequences (>700 points). Next, MS-RP images are obtained by multiplying designed sign masks in order to remove the tendency confusion. Finally, FCN is trained with MS-RP images to perform classification. Experimental results on 45 benchmark datasets demonstrate that our method improves the state-of-the-art in terms of classification accuracy and visualization evaluation.

## 1. Introduction

In the era of big data, the real world produces a huge number of time series worth being analyzed. Among all the time series analyzing tasks, classification is likely to be the most fundamental one, which predicts the associated category labels of sequences to be investigated. Due to the development and maturity of sensor technology, time series classification (TSC) problems arise across a wide range of domains, e.g., action recognition, medical diagnosis, natural language processing, mechinery fault diagnosis, electrical energy monitoring [1,2,3], etc., and have received more and more attention.

In the literature, TSC approaches fall into three popular categories: feature based, distance based and ensemble based. Feature-based approaches extract representative features from time series, and then use a classifier to map each of them to a category [4,5,6,7,8,9,10]. Distance-based approaches measure the elastic distance between the testing and training set, and assign a label to the testing based on the distance similarity [11,12,13,14,15]. Ensemble based methods integrate different features and multiple classifiers in one framework, thus obtaining a complementary effect and better classification accuracy [16,17]. Recent years have witnessed the great success of deep neural networks (DNN) in various domains. In particular, DNN-based methods have also been explored in TSC and show inspiring advancement. Reference [18] proposes a multi-scale Convolutional Neural Network (CNN), which first preprocesses the raw data through down-sampling, smooth filtering, and slicing up to perform data augmentation; then, a traditional CNN is applied. Reference [19] proposes Multilayer Perceptrons (MLP), Fully Convolutional Networks (FCN), and Residual Networks (ResNet) as baseline architectures for TSC, which are the most traditional forms of DNNs. It is worth mentioning that FCN and ResNet are regarded as the best DNN-based classifiers for TSC [3]. Reference [20] proposes a multilevel Wavelet Decomposition Network, which first decomposes a time series into different frequencies of subsequences through a fine-tuned Daubechies 4 Wavelet; these subsequences are then handled with FCN and ResNet for classification. This work achieves very strong performance among existing DNN-based methods. Reference [21] augments FCN with Attention LSTM (ALSTM) module. ALSTM exhibits temporal information and obviously supplements the performance of FCN. This work achieves state-of-the-art accuracy among existing DNN-based methods.

To make better use of the outstanding classification power of CNN, some recent works first encode time-series as images, and then transform TSC problem to image classification. With this way, the distinctive regions of a sequence are magnified and the temporal correlations are constructed, thus an improvement in accuracy can be achieved. References [22,23,24] assemble Gramian Angular Fields (GAF) and Markov Transition Fields (MTF) of sequences in multi-channel images, then train a Tiled CNN on these images for classification. Reference [25] transforms the acceleration data and angular velocity data to multi-channel GAF images respectively, and then utilizes a multi-branch residual network to fuse these images for human activity recognition. Similarly, reference [26] encodes time series as images through Recurrence Plots (RP), and then trains a CNN on RP images to perform classification. This work achieves best performance among representative sequence-to-image methods, which raises the interest of the community in it.

In this paper, inspired by the rich texture information provided by RP and the outstanding results of DNN in image classification [27,28,29], we incorporate RP images and the state-of-the-art DNN classifiers of TSC in one framework. RP is a widely used visualization technique for analyzing dynamical systems [30,31]. Due to the graphical nature of exposing hidden patterns and local correlation information of a sequence, RP has been introduced to TSC for representing time series as images [26,32,33]. However, several defects limit its further application in TSC. In this paper, we summarize three major challenges and provide our solutions as follows.

Firstly, sequences of different datasets vary significantly in length and their distinctive regions usually distribute on various scales. Existing methods deal with this problem only by adjusting the image size [26,32,33,34]. However, to avoid high computational overhead, the adjustable size is limited to a small range, which often decreases the representation ability of RP. We address this challenge by additionally introducing phase space dimension (*m*) and embedding time delay (τ) of RP, which are fixed in other methods [26,32,33,34]. Different values of *m*, τ, and image sizes are explored according to different datasets in order to enrich the scales of RP images. The images with suitable scales will be selected as the input to a classifier.

Secondly, RP is not good at encoding very long sequences, especially when the length of a sequence is larger than 700. For very long sequences, the sizes of their RP images are so large that they are downsampled to being small enough. Consequently, it causes the loss of image information. We address this challenge by constructing asymmetrical RP images. Specifically, a sequence is first divided into two pieces, and each piece is encoded as an RP image. Then, thanks to the symmetrical structure of RP image, the oblique triangle matrix of each RP image is extracted and reassembled into one image. By this way of constructing an asymmetric matrix, the information loss caused by downsampling obviously alleviates.

Thirdly, RP confuses the tendencies of time series. The reason is that the norm operation, being used to calculating the distances of states in the phase space, leaves all of the pixel values in RP images positive. Thus, RP could not distinguish the rising and falling trend of sequences. We address this challenge by introducing the rule of signs. Specifically, designed signed masks are calculated, which utilize the positive and negative values to indicate the rising and falling trend of the sequences. Then, these masks are multiplied to RP images for supplementing trend changing information.

We incorporate the aforementioned solutions together to propose Multi-scale Signed Recurrence Plots (MS-RP), then FCN and ResNet, which are the state-of-the-art classifiers of TSC, are applied to classify MS-RP images. Compared with the state-of-art time series classification algorithms, the advantages of this algorithm are mainly reflected in two aspects. First, the proposed MS-RP preserves the advantages of RP in providing temporal correlations and magnifying the distinctive regions. Moreover, compared with other image encoding algorithms, MS-RP better accommodates the variations of sequences in tendency, length, and scale. Second, the state-of-the-art deep learning classifiers, FCN, and ResNet, are used to handle the transformed MS-RP images, which further improves classification performance.

Our proposed method achieves superior performance in 45 UCR (University of California, Riverside) time series classification datasets [35] and the validation experiments are provided hierarchically. Moreover, utilizing t-Distributed Stochastic Neighbor Embedding (t-SNE) [36], we visualize the spatial distribution of the latent representation learned by the networks. It clearly demonstrates that MS-RP better utilizes the advantage of DNN in extracting features.

## 2. Approaches

**Figure 1:**
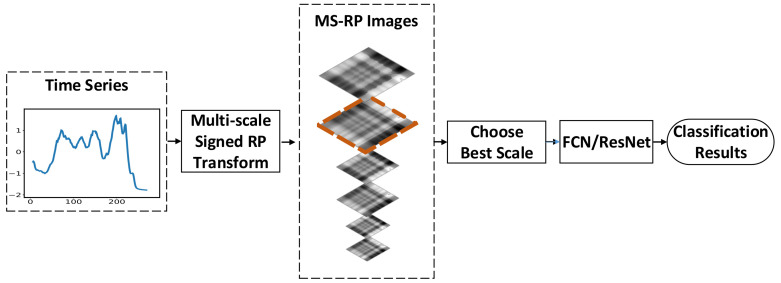
The framework of our approach. The *x*-axis and *y*-axis of time series represent the length and the amplitude, respectively.

The proposed approach in this paper consists of two stages. In the first stage, we improve RP comprehensively into MS-RP, and encode time series as MS-RP images. In the second stage, FCN and ResNet are applied to handle these images. The framework of our approach is shown in Figure 1.

As shown in the figure, the input time series are first transformed into images through MS-RP. Then, the MS-RP images are produced in various scales, and the images with best scale are selected. Finally, the selected images are taken as the input of FCN and ResNet classifiers instead of the original sequences for classification.

### 2.1. Proposed MS-RP

**Figure 2:**
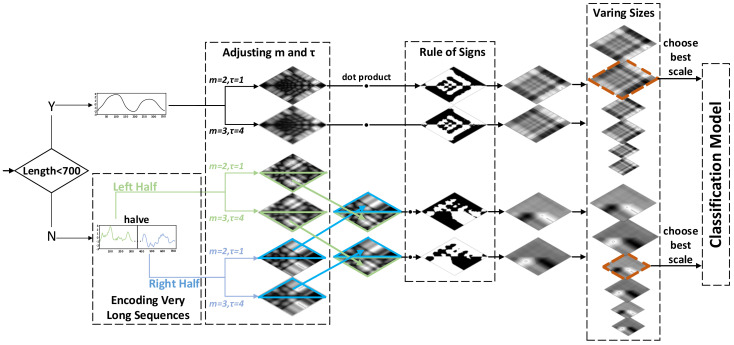
The architecture of our proposed MS-RP. The *x*-axis and *y*-axis of time series represent the length and the amplitude, respectively.

In this section, MS-RP is introduced in four parts. The basic theory of RP is described in Section 2.1.1, the process of multi-scale RP is described in Section 2.1.2, Section 2.1.3 illustrates how to encode very long sequences, and Section 2.1.4 introduces the rule of signs for RP. The general overview of MS-RP is shown in Figure 2.

As shown in the figure, we separate the input sequences into two cases. For short sequences (less than 700 in length), a sequence is first encoded as RP images in two different scales, and the sign masks are extracted and multiplied to these RP images. Then, these multi-scale signed RP images are resized into multiple sizes. Finally, through classification performances on the validation sets, the image with the best scale is selected and taken as the input instead of the original sequences for classification. For long sequences (more than 700 in length), a sequence is first divided into two pieces of equal length, and each piece is encoded as RP images just like a short sequence. Then, utilizing the symmetrical structure of RP, two RP images corresponding to two divided pieces are reassembled in one asymmetrical image. The rest part of the encoding process stays consistent with short sequences.

#### 2.1.1. Review of RP

**Figure 3:**
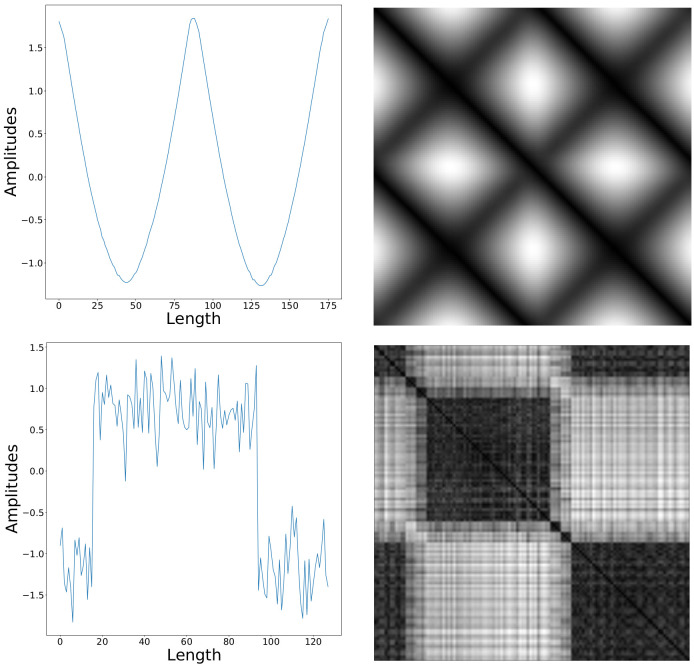
RP images of different sequences.The sequences come from ‘Adiac’ dataset (upper row) and ‘CBF’ dataset (lower row), respectively.

RP is a visualization tool widely used to analyze the recurrent behaviors of time series generated in dynamical systems [30,31]. Concretely, a sequence is mapped to m-dimensional phase space; then, RP image of the sequence is achieved by calculating the distance matrix between the states in the phase space. RP reveals the local correlation information of a sequence through distance calculation between subsequences, while autocorrelation information is crucial to TSC [16]; thus, it is widely used to encode sequences as images (see Figure 3). Equation (1) defines RP formally:

(1) $$\begin{array}{cc} RP_{i,j}\left(\epsilon \right)= & \Theta (\epsilon -∥\vec{x}\left(i\right)-\vec{x}\left(j\right)∥),\\ \; & \vec{x}\left(\cdot \right)\in R^{m},i,j=1,\ldots ,N \end{array}$$

where *N* is the number of states, *m* is the phase space dimension, $\vec{x}\left(i\right)$ is *i*-th state in the phase space as well as the subsequence observed at the *i*-th position of the sequence, ∥·∥ is a norm operation, ϵ is a threshold, Θ is the Heaviside function used to binarize the distance matrixes, whose value is zero for negative argument and one for positive argument, and $RP_{i,j}$ is the pixel at position (i,j) of the RP image. Moreover, another important parameter for the generation of states is embedding time delay τ, which can be regarded as the sampling interval of the time series. Actually, the binarization step is usually omitted in TSC, to avoid texture information loss; thus, Equation (1) can be simplified into Equation (2):

(2) $$RP_{i,j}\left(\epsilon \right)=∥\vec{x}\left(i\right)-\vec{x}\left(j\right)∥,\vec{x}\left(\cdot \right)\in R^{m},i,j=1,\ldots ,N$$

Though RP provides rich texture information [32,34] and facilitates the application of convolutional networks [26,37], the mentioned challenges of Section 1 limit its further application in TSC. In the following sections, the improvements of RP will be illustrated in detail.

#### 2.1.2. Multi-Scale RP: An Improvement of RP

The distinctive regions of sequences appear in various scales, and the lengths of sequences vary largely. Existing methods adapt to these variabilities through adjusting the image sizes. However, considering the computing costs, these image sizes are controlled in a relatively small range, thus limiting the representation ability of RP.

The generation process of RP images is similar to the process of dilated convolution operations. The subsequences sliding over the raw data can be regarded as dilated convolution kernels, except that norm calculation is replaced by inner product. The lengths and sampling intervals of the subsequences correspond to the kernel sizes and dilatation rates, respectively, and can be controlled by *m* and τ. Different values of *m* and τ vary the receptive fields of sliding subsequences, and temporal correlations can be constructed in various scales.

Thus, phase space dimension *m* and embedding time delay τ of RP are introduced to address this challenge, which are always ignored and kept fixed in other articles. According to different datasets, the values of *m* and τ are adjusted together with image sizes to produce multi-scale images. Through selecting the multi-scale images properly, temporal correlations can be built in suitable scales, and image sizes can better adapt to the length variability of sequences as well as the receptive field of the network.

**Figure 4:**
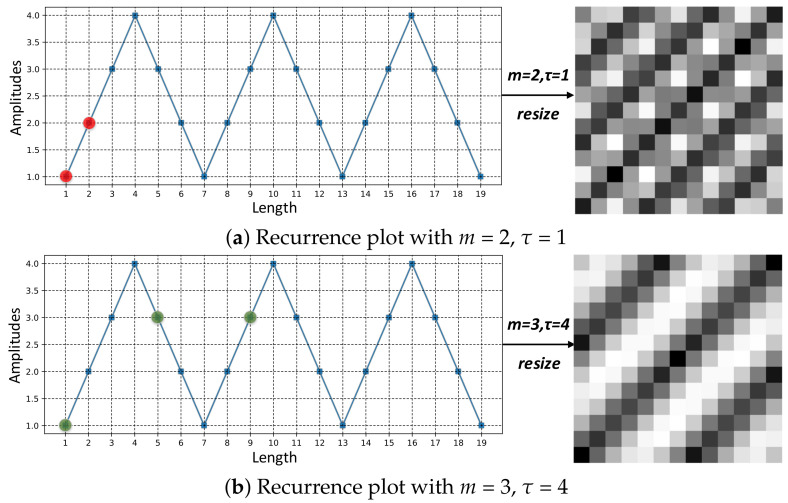
The subsequences and RP images of a triangular periodic sequence, with different values of *m* and τ. The red and blue dots in (**a**,**b**) correspond to the first subsequences of the sequence when (m,τ) are (2,1) and (3,4), respectively.

The most commonly used values of (m,τ) are either (2,1) or (3,4) [26,32,33,34]. Both of them are adopted in this paper, corresponding to two different scales of RP images. Such a small search scope of (m,τ) is due to our initial motivation validating the significance of adjusting these two parameters, other than searching for the best values. Figure 4 shows a triangle periodic sequence and its RP images with these two groups of *m* and τ. It can be seen that, even with same image sizes, smaller values of (m,τ) produce a more fine-grained image, while larger values of (m,τ) produce an image with overall information.

#### 2.1.3. Asymmetrical RP for Encoding Very Long Sequences

For very long sequences (>700), the sizes of RP images can be very large. On the one hand, RP images with such large sizes will bring computation explosion, on the other hand; if these images are resized to reasonable sizes, it will lead to serious information loss.

**Figure 5:**
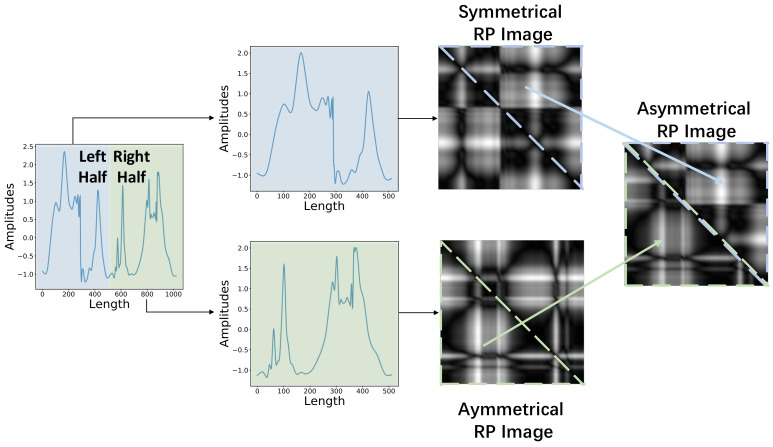
The illustration of constructing an asymmetrical RP image (the sequence comes from ‘Mallat’ dataset).

To address this challenge, asymmetrical RP is proposed. Figure 5 shows the process of constructing an asymmetrical RP image. As is shown, a sequence is halved into two pieces, and each piece is encoded as an image. Then, the upper and lower oblique triangle matrixes of the two images are extracted separately and then reassembled in one image, utilizing the symmetrical structure of RP. Through constructing the asymmetrical RP images, we alleviate the information loss brought in the resizing process, and overcome the information redundancy problem of symmetrical RP.

#### 2.1.4. Rule of Signs

As is indicated in Equation (2), norm operation is utilized for the distance calculation between states in the phase space, these distances correspond to the pixel values in the RP image. The commonly used norm operations are $L_{1}-norm$, $L_{2}-norm$ and $L_{\infty }-norm$; however, no matter which norm operation is selected, all of the pixel values of RP images are positive, leading to serious tendency confusion problem of RP.

A simple example can illustrate this problem. $s_{1}$ and $s_{2}$ are two short sequences, whose values are [1,2,3] and [3,2,1], corresponding to two opposite monotonous trends, respectively. Equation (2) is utilized for the calculation of RP matrixes, where ∥·∥ is $L_{2}-norm$ and (m,τ) is (2,1). The RP matrixes of $s_{1}$ and $s_{2}$ are shown in Equations (3) and (4) separately. As is shown, the RP matrixes of these two sequences are totally the same:

(3) $$RP_{s_{1}}=\left(\begin{array}{cc} 0 & \sqrt{2}\\ \sqrt{2} & 0 \end{array}\right),$$

(4) $$RP_{s_{2}}=\left(\begin{array}{cc} 0 & \sqrt{2}\\ \sqrt{2} & 0 \end{array}\right).$$

**Figure 6:**
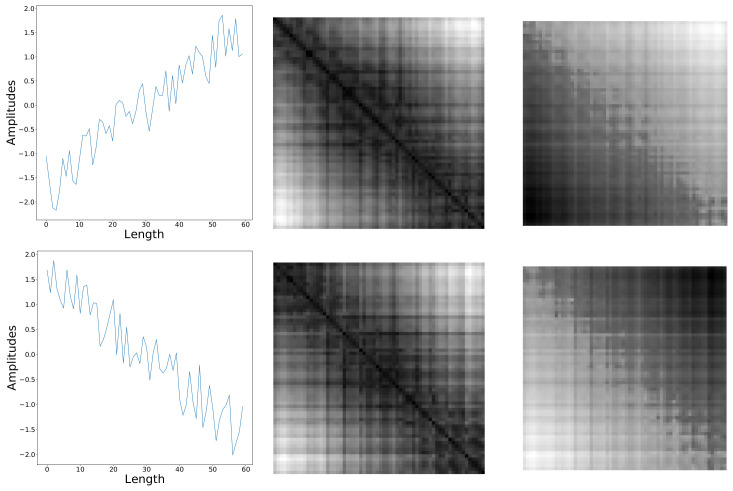
The RP images of sequences with opposite tendencies. (left column: two sequences with opposite tendencies from ’SyntheticControl’ dataset, middle column: the RP images of the sequences, right column: signed RP images of the sequences).

To address this challenge, the rule of signs is introduced for RP. Firstly, a sequence is mapped to phase space, then the subtraction and norm operations between states in phase space are performed, to obtain the state difference vectors and the RP image pixel values respectively. Secondly, we sum each state difference vector separately; then, the signs of the sum values are extracted to construct a sign mask with the same size of the RP image. Finally, the sign mask is multiplied to the RP image, thus we obtain the signed RP image. The whole process is defined by Equation (5):

(5) $$\begin{array}{cc} RP_{i,j}\left(\epsilon \right)= & \frac{sum\left(\vec{x}\left(i\right)-\vec{x}\left(j\right)\right)\cdot ∥\vec{x}\left(i\right)-\vec{x}\left(j\right)∥}{\left|sum(\vec{x}\left(i\right)-\vec{x}\left(j\right))\right|},\;\\ \; & \vec{x}\left(\cdot \right)\in R^{m},i,j=1,\ldots ,N \end{array}$$

where sum is the vector summation function, ∥·∥ is $L_{2}-norm$, |·| is the function calculating absolute values. As a visual illustration, Figure 6 (left) shows the RP images of two sequences with opposite tendencies. These sequences come from ’SyntheticControl’ dataset. Figure 6 (middle) shows the RP images of the two sequences, and they can hardly be distinguished. Figure 6 (right) shows the signed RP images of the two sequences, and these signed images reflect the trend of sequences and can be easily distinguished.

### 2.2. Classification Using FCN on MS-RP Images

In the last section, RP is modified comprehensively into MS-RP to encode time series as images. A high performance classifier should be applied for these images. Existing methods usually combine RP with k-nearest neighbor (kNN) classifiers [32,34] or traditional CNN classifiers [26,37]. However, the performances of kNN classifiers are heavily dependent on the handcrafted features; in addition, although the traditional CNN classifiers unify feature learning and classification in one framework, the pooling operation leads to serious information loss, and the fully connected layers with huge number of parameters may overfit the MS-RP images.

**Figure 7:**
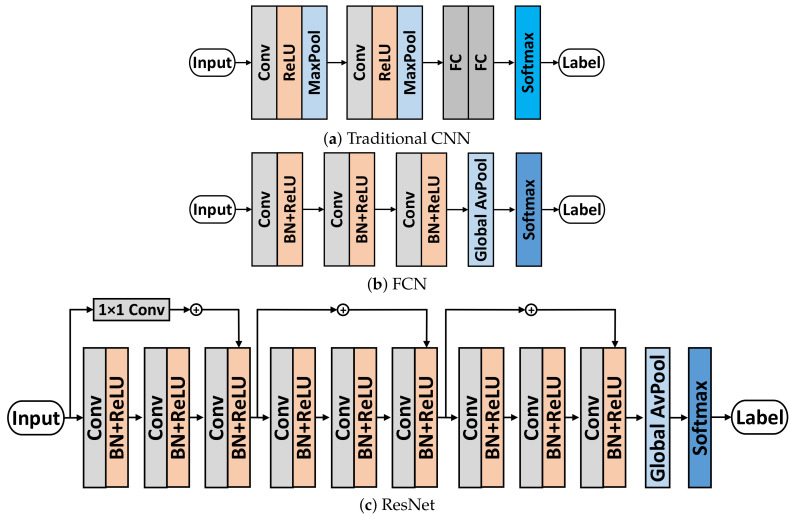
The architectures of network classifiers used in this paper.

To address these problems, in this paper, FCN and ResNet are introduced to handle MS-RP images, which are expanded to 2D-version according to the image data format. FCN and ResNet are firstly proposed as baseline classifiers in [19], and they are widely regarded as the state-of-the-art classifiers for TSC [3]. The architectures of these two networks are shown in Figure 7b,c. FCN is a fully convolutional network, which has three convolution layers; each layer follows a Batch Normalization (BN) layer and a Rectified Linear Unit (ReLU) activation function. FCN has no Fully Connected (FC) layers. After the convolution process, the features pass through a Global Average Pooling layer and a Softmax layer for classification. ResNet expands FCN through residual connections. It has three residual blocks, and each block has the same structure with FCN. ResNet explores a network with deeper architecture; it is a compromise for balancing better representations and overfitting.

## 3. Experiments and Analysis

### 3.1. Experimental Setup

Our proposed method and the state-of-the-art competitors are evaluated on 45 datasets of the UCR archive, which is an assembly of TSC datasets coming from various domains in the real world [35]. The competitive approaches are listed as follows:

- FCN and ResNet: These two models are proposed in [19], which have been regarded as the strong baseline and best DNN-based classifiers for TSC.
- RP-CNN: [26] combines RP with a traditional CNN, which is similar to our proposed approach. We take it as the baseline for methods encoding time series as images. The RP image sizes are consistent with our approach for fairness, and (*m*, τ) of RP are (3, 4). The architecture of traditional CNN is shown in Figure 7a. The traditional CNN consists of two convolutional layers (32 channels, 3 × 3 kernel), two pooling layers (2 × 2 Max Pooling), two FC layers (125 neurons), and a Softmax layer.
- RP-FCN: This model combines RP and FCN into one frame work. It is provided by us for comparison between RP and MS-RP.
- HIVE-COTE: This model ensembles five different features with various heterogeneous classifiers [17]; it achieves state-of-the-art performance among traditional time series classification methods.
- FCN Residual Classification Flow (FCN-RCF): [20] decomposes sequences as multiple frequencies of subsequences through fine-grained wavelet, and FCN are then applied to handle these subsequences. This model achieves very strong performances.
- ALSTM-FCN: This model combines FCN and ALSTM in one framework [21]. ALSTM supplements important temporal information for FCN, which obviously improves classification performance. The proposed ALSTM-FCN achieves state-of-the-art performance among DNN based methods.

The adjustable parameters of MS-RP are *m*, τ and image sizes, which vary according to different dataset. The values of (m,τ) are selected between (2,1) and (3,4), and the image sizes range over (16,48,64,80,96,112,128). Suitable parameters can be obtained according to classification performances on the validation set. We first initialize the image sizes (usually 64, larger or smaller according to the sequence length), and search the values of (*m*, τ). Then, we fix (*m*, τ) and search the image sizes from the aforementioned range scope. Note that the search scope of image sizes can be narrowed according to the sequence length. For the network parameter configuration, the sizes and channel numbers of convolutional kernels in FCN are given in Table 1. ResNet is expanded from FCN, thus the parameters of each residual block in ResNet stay consistent with FCN. The MS-RP image sizes are provided in Table 2, and the three numbers in the parentheses represent the values of image sizes, *m* and τ, respectively. FCN and ResNet are trained utilizing “categorical-crossentropy” loss function and ’Adam’ optimizer [38], with learning rate 5e−5.

The classification results of our proposed approach are the average of five repeated experiments. The performances of the competitors are directly obtained from the corresponding articles [17,19,21,26], and we supplement the missing experimental results utilizing author provided code. For the evaluation of our proposed approach and the competitors, Number of Wins, Average Arithmetic ranking, Average Geometric ranking, and Mean Per-Class Error (MPCE) are introduced from [19]. Then, we follow the recommendations of [39] to adopt the Friedman test for rejecting the null hypothesis [40]. Finally, utilizing a Wilcoxon signed-rank test with Holm correction (α = 0:05) [41,42], we measure the significance of difference between different classifiers. A critical difference (CD) diagram [39] is performed to visualize these comparisons intuitively.

### 3.2. Results and Analysis

**Figure 8:**
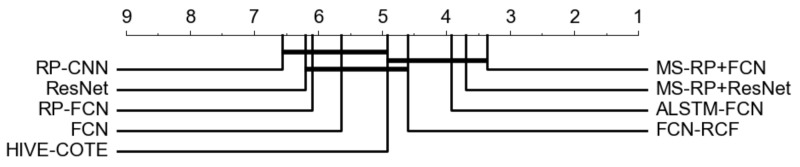
The CD diagram of the competitive approaches and our proposed two classifiers over 45 UCR datasets; the thick horizontal lines in the diagram indicate a cluster of classifiers that are not significantly different in terms of classification performance.

**Figure 9:**
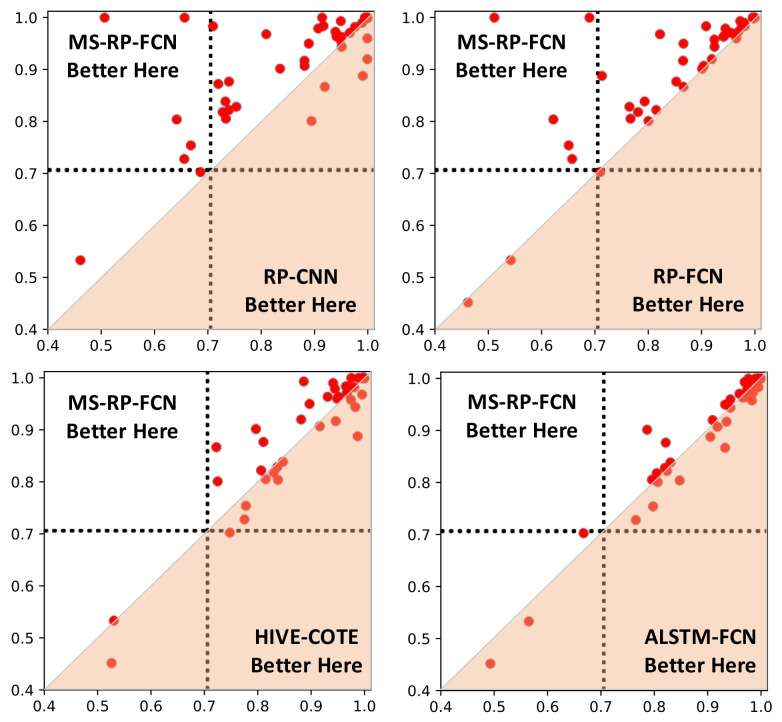
Pairwise comparison of classification performances between MS-RP-FCN and the competitors.

Comparison of classification results is listed in Table 2, with the best performance of each dataset highlighted in bold. The CD diagram is shown in Figure 8. Moreover, pairwise comparison between MS-RP-FCN and its competitors are provided in Figure 9.

In the CD diagram of Figure 8, it is clear to see that MS-RP-FCN and MS-RP-ResNet achieve the best performance among all of the competitors. The evaluation indexes of Table 2 shows that MS-RP-FCN wins three of the four metrics and MS-RP-ResNet wins two of the four metrics. For the MPCE index, MS-RP-FCN and MS-RP-ResNet are tied for the first. For the Arithmetic ranking and Geometric ranking indexes, MS-RP-FCN ranks first and MS-RP-ResNet ranks second. The relative disadvantage of MS-RP-FCN is win number index. This is due to the sizes of datasets in the UCR archive having a large variability. FCN is slightly inferior to large datasets due to the shallow structure.

Some interesting and more detailed observations can be made as follows. First, compared with FCN and ResNet, the advantages of our proposed methods are obvious. It demonstrates that the texture information provided by MS-RP can be more easily distinguished by the networks. Second, the performances of MS-RP-FCN are far better than RP-CNN and RP-FCN, due to better classifiers and the improvement of RP. Finally, although HIVE-COTE, FCN-RCF, and ALSTM-FCN achieve very competitive performances, they have small gaps with MS-RP-FCN and MS-RP-ResNet as shown in Figure 8, which further demonstrates the effectiveness of our proposed methods.

Considering MS-RP is composed of three parts as mentioned in Section 2.1, and the effectiveness of each part should be demonstrated. Thus, the validation experiment of each part is provided in Table 3, Table 4 and Table 5 respectively, with FCN selected as the classifier. For visual convenience, the best performance of each dataset in these tables is highlighted in bold.

**Comparison between Different Values of m and τ.**Table 3 provides ten pairs of classification error rates for performance comparison between two different groups of *m* and τ, with the image sizes staying consistent with Table 2. Distinct gaps between the two groups of error rates can be found in the table. As is aforementioned, different values of (m,τ) enrich the scales of RP images, which are helpful in better representing time series.

**Comparison between Symmetric RP and Asymmetric RP.** Asymmetric RP images are proposed for encoding very long sequences. To compare the performances of symmetric and asymmetric RP, six UCR datasets with very long sequences are selected, and the error rates are provided in Table 4. It can be seen that the asymmetric structure is helpful, though the gains are small except for the ’CinCECGTorso’ dataset. This is likely due to asymmetric RP being more capable of preserving detailed information, while most selected datasets own global shapes.

**Comparison between Signed RP and Unsigned RP.** The rule of signs is introduced to overcome the tendency confusion problem of RP. To evaluate its effectiveness, we select ten datasets and provide the performances of signed and unsigned RP in Table 5. As is shown, signed RP has obtained a huge advantage. Thus, the sign masks are effective supplements to RP, which describe the tendency variations of sequences. Actually, the sign masks are more critical for action recognition datasets, and sequences of these datasets are more sensitive to tendency changing.

### 3.3. Visualization

In order to visually demonstrate that MS-RP better utilizes the advantage of DNN in extracting features, we gain some insights on the spatial distribution of the latent representation learned by the networks. Specifically, we feed the raw data, RP images, and MS-RP images into FCN respectively, and extract the last latent representations (feature vectors of global average pooling layer) learned by the network. Then, t-Distributed Stochastic Neighbor Embedding (t-SNE) [36] is introduced to visualize the classification results of different input data. It is a technique embedding high-dimensional vectors into a two-dimensional map.

**Figure 10:**
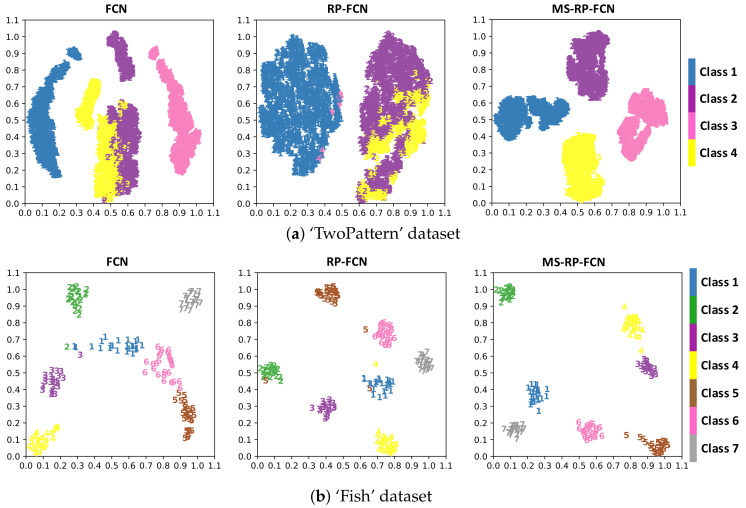
Visualizations of different input data by t-SNE. (left column: features learned from the raw data; middle column: features learned from the RP image data; right column: features learned from MS-RP image data).

We select ’TwoPatterns’ and ’Fish’ datasets to produce the mentioned three kinds of input data, and FCN is trained on 2000 epochs with each kind of data. Figure 10 shows the visualization results. As is shown, when FCN is trained with the raw data, the feature crowds of different classes are hard to separate, and they are close in distance. When FCN is trained with RP images, the results of the ’Fish’ dataset are pretty good, though small category confusion still exists, while the results of ’TwoPattern’ dataset are disastrous, due to the tendency confusion problem of RP. When FCN is trained with MS-RP images, the feature crowds of different classes can be totally distinguished with a relatively large distance on both datasets.

## 4. Conclusions

In this paper, we improve RP comprehensively into MS-RP, and then transform TSC problems as image classification tasks for DNN. Firstly, phase space dimension *m* and embedding time delay τ of RP are introduced to enrich the scales of RP images. Secondly, asymmetrical RP is constructed to encode very long sequences. Finally, the rule of signs is introduced to overcome the tendency confusion problem of RP. Moreover, FCN and ResNet are trained to handle MS-RP images, which are state-of-the-art classifiers for TSC.

Experimental results on 45 UCR datasets demonstrate that our proposed method outperforms the state-of-the-art, and each block of MS-RP is also demonstrated hierarchically through validation experiments. Furthermore, utilizing t-SNE, the classification results of different input data are analyzed visually, which further supports the effectiveness of our proposed approach.

Thanks to the the popularity of wearable sensors, our work can be easily extended to practical applications, e.g., motion recognition, ECG health, and sleep state monitoring on mobile phones. We would like to take these interesting jobs as our future work. 

## Figures and Tables

**Table 1:** Parameter configuration of FCN.

Network Parameters	Convolution Blocks		
	Conv1	Conv2	Conv3
Conv Kernel Size	5 × 5	5 × 5	5 × 5
Filter Channel Number	128	256	128

**Table 2:** Comparison in terms of classification error rates on 45 UCR datasets.

Dataset	RP-CNN	Res-Net	FCN	RP-FCN	HIVE-COTE	FCN-RCF	ALSTM-FCN	MS-RP-Res	MS-RP-FCN
Adiac(64,2,1)	0.2800	0.1740	0.1430	0.1709	0.1846	0.1550	**0.1330**	0.1560	0.1379
Beef(64,2,1)	0.0800	0.2330	0.2500	0.1667	0.2773	**0.0300**	0.0667	0.0667	0.1333
CBF(64,2,1)	0.0050	0.0060	**0**	0.0033	0.0006	**0**	**0**	**0**	**0**
ChlorineCon(112,2,1)	0.1049	0.1720	0.1570	0.1992	0.2749	**0.0680**	0.1930	0.1987	0.1992
CinTorso(128,3,4)	**0.0087**	0.2290	0.1870	0.2866	0.0120	0.0140	0.0942	0.0486	0.1123
Coffee(64,2,1)	**0**	**0**	**0**	**0**	0.0018	**0**	**0**	**0**	**0**
CricketX(64,3,4)	0.2718	0.1790	0.1850	0.2187	0.1696	0.2160	0.1949	**0.1538**	0.1821
CricketY(64,3,4)	0.2462	0.1950	0.2080	0.2349	0.1630	0.1720	0.1795	**0.1564**	0.1718
CricketZ(64,3,4)	0.2667	0.1870	0.1870	0.2064	0.1523	0.1620	0.1692	**0.1487**	0.1615
DiatomSizeR(64,2,1)	0.0098	0.0690	0.0700	0.0196	0.0581	0.0230	0.0261	**0.0065**	0.0098
ECG200(64,2,1)	**0**	0.1300	0.1000	0.0800	0.1181	0.0625	0.0900	0.0500	0.0800
ECGFiveDays(64,3,4)	0.0023	0.0450	0.0150	**0**	0.0105	0.0100	0.0046	**0**	**0**
FaceAll(96,2,1)	0.1900	0.1660	0.0710	0.1775	**0.0037**	0.0980	0.0343	0.0627	0.0320
FaceFour(96,2,1)	**0**	0.0680	0.0680	0.0341	0.0505	0.0500	0.0568	0.0795	0.0400
FacesUCR(64,2,1)	0.0483	0.0420	0.0520	0.0751	**0.0164**	0.0870	0.0566	0.0585	0.0561
FiftyWords(48,3,4)	0.2600	0.2730	0.3210	0.1846	0.1932	0.2880	0.1758	**0.1692**	0.1780
Fish(64,2,1)	0.0850	0.0110	0.0290	**0**	0.0238	0.0210	0.0229	0.0114	**0**
GunPoint(64,2,1)	**0**	0.0070	**0**	**0**	0.0033	**0**	**0**	**0**	**0**
Haptics(64,2,1)	0.5390	0.4940	0.4490	0.4578	0.4697	0.4610	**0.4351**	0.4708	0.4675
InlineSkate(128,2,1)	0.6436	0.6350	0.5890	0.5382	**0.4741**	0.5660	0.5073	0.5655	0.5491
ItaPowDemand(16,2,1)	0.0330	0.0400	0.0300	0.0447	0.0322	0.0310	0.0398	**0.0262**	0.0292
Lightning2(64,3,4)	0.1639	0.2460	0.1970	**0.0984**	0.2030	0.1450	0.2131	0.1148	**0.0984**
Lightning7(64,3,4)	0.2600	0.1640	0.1370	0.1470	0.1889	**0.0910**	0.1781	0.1440	0.1233
Mallat(128,3,4)	0.0512	0.0210	0.0200	0.0752	0.0245	0.0440	**0.0162**	0.0473	0.0422
MedicalImg(96,2,1)	0.2658	0.2280	0.2080	0.2329	0.1846	**0.1640**	0.2039	0.2066	0.1947
MoteStrain(80,2,1)	0.1182	0.1050	**0.0500**	0.1741	0.0532	0.0760	0.0639	0.0847	0.0831
NonInThorax1(128,3,4)	0.0580	0.0520	0.0390	0.0580	0.0683	0.0260	**0.0249**	0.0575	0.0361
NonInThorax2(128,3,4)	0.0489	0.0490	0.0450	0.0579	0.0477	**0.0280**	0.0336	0.0453	0.0366
OliveOil(96,2,1)	0.1100	0.1330	0.1670	0.1333	0.1023	**0**	0.0667	0.0667	0.0500
OSULeaf(96,2,1)	0.2900	0.0210	0.0120	0.0909	0.0295	0.0180	**0.0041**	0.0248	0.0165
SonyAIRobot1(64,2,1)	0.0499	0.0150	0.0320	0.0266	0.1132	0.0420	0.0300	0.0166	**0.0067**
SonyAIRobot2(64,2,1)	0.0923	0.0380	0.0380	0.0546	0.0546	0.0640	0.0252	0.0535	**0.0210**
StarLigCurves(128,3,4)	0.0234	0.0250	0.0330	0.0238	0.0185	0.0180	0.0233	0.0195	**0.0180**
SwedishLeaf(64,2,1)	0.0600	0.0420	0.0340	0.0304	0.0314	0.0570	**0.0144**	0.0272	0.0272
Symbols(64,3,4)	0.0824	0.1280	0.0380	0.0181	0.0342	0.0400	**0.0131**	0.0141	0.0161
SynControl(64,2,1)	0.3433	**0**	0.0100	0.3100	0.0004	0.0382	0.0100	**0**	**0**
Trace(64,2,1)	**0**	**0**	**0**	**0**	**0**	0.0940	**0**	**0**	**0**
TwoLeadECG(64,2,1)	0.0026	**0**	**0**	0.0018	0.0065	0.0643	0.0009	**0**	**0**
TwoPatterns(64,2,1)	0.4935	**0**	0.1030	0.4888	0.0001	**0**	0.0032	**0**	**0**
UWaveX(64,3,4)	0.3582	0.2130	0.2460	0.3778	0.1616	0.2180	**0.1519**	0.1790	0.1963
UWaveY(64,3,4)	0.3439	0.3320	0.2750	0.3425	**0.2245**	0.2320	0.2342	0.2496	0.2725
UWaveZ(64,2,1)	0.3317	0.2450	0.2710	0.3490	0.2217	0.2650	**0.2018**	0.2272	0.2462
Wafer(64,2,1)	**0**	0.0030	0.0030	0.0015	0.0003	**0**	0.0019	0.0006	0.0011
WoSynonyms(64,2,1)	0.3135	0.3680	0.4200	0.2900	**0.2520**	0.3380	0.3323	0.2774	0.2978
Yoga(64,2,1)	0.1180	0.1420	0.1550	0.0953	0.0830	0.1120	**0.0810**	0.0887	0.0930
Win num	7	5	6	6	6	11	**14**	**14**	13
Arithmetic ranking	6.3111	5.9111	5.2889	5.7778	4.8444	4.3778	3.6444	3.3111	**2.9111**
Geometric ranking	5.0800	4.9744	4.3512	4.7974	3.8710	3.4003	2.8214	2.6007	**2.4147**
MPCE	0.0256	0.0240	0.0220	0.0252	0.0203	0.0175	0.0175	**0.0164**	**0.0164**

**Table 3:** Comparison in terms of error rates between different *m* and τ.

Dataset	Adiac	Face- All	Medical- Img	Mote- Strain	OSU- Leaf	CricketY	CricketZ	Fifty- Words	Lightning2	Lightning7
*m* = 2,τ = 1	**0.1379**	**0.0320**	**0.1947**	**0.0831**	**0.0165**	0.2077	0.1897	0.2066	0.1475	0.1507
*m* = 3,τ = 4	0.1637	0.0698	0.2316	0.1222	0.0620	**0.1718**	**0.1615**	**0.1780**	**0.0984**	**0.1233**

**Table 4:** Comparison in terms of error rates between symmetric RP and asymmetric RP.

Dataset	CinTorso	InlineSkate	Mallat	NonInThorax1	NonInThorax2	StarLigCurves
Asymmetric RP	**0.1123**	0.5491	**0.0422**	**0.0361**	**0.0366**	**0.0180**
Symmetric RP	0.2866	**0.5382**	0.0729	0.0539	0.0514	0.0232

**Table 5:** Comparison in terms of error rates between signed RP and unsigned RP.

Dataset	CricketX	CricketY	CricketZ	Lightning7	OSU- Leaf	Syn- Control	Two- Patterns	UWaveX	UWaveY	UWaveZ
Signed RP	**0.1821**	**0.1718**	**0.1615**	**0.1233**	**0.0165**	**0**	**0**	**0.1963**	**0.2725**	**0.2462**
Unsigned RP	0.2187	0.2349	0.2064	0.1469	0.0744	0.2967	0.4850	0.3778	0.3425	0.3431
